# Right ventricular stiffness as a key feature in the ZSF1 model of heart failure with preserved ejection fraction

**DOI:** 10.1093/ehjimp/qyag027

**Published:** 2026-02-13

**Authors:** Florian Schlotter, Karl-Patrik Kresoja, Karl-Philipp Rommel, Lena Rosenbusch, Sarah Werner, Urvashi Sharma, Holger Thiele, Christian Besler, Philipp Lurz, Petra Büttner

**Affiliations:** Department of Cardiology, University Medical Center of the Johannes Gutenberg University Mainz, Langenbeckstr. 1, 55131 Mainz, German Center for Cardiovascular Research - Partner Site Rhine-Main; Department of Cardiology, Heart Center Leipzig at Leipzig University, Leipzig, Germany; Department of Cardiology, University Medical Center of the Johannes Gutenberg University Mainz, Langenbeckstr. 1, 55131 Mainz, German Center for Cardiovascular Research - Partner Site Rhine-Main; Department of Cardiology, Heart Center Leipzig at Leipzig University, Leipzig, Germany; Department of Cardiology, University Medical Center of the Johannes Gutenberg University Mainz, Langenbeckstr. 1, 55131 Mainz, German Center for Cardiovascular Research - Partner Site Rhine-Main; Department of Cardiology, Heart Center Leipzig at Leipzig University, Leipzig, Germany; Clinic for Internal Medicine III, Universitätsmedizin Halle, Universitätsklinikum Halle, Halle (Saale), Germany; Department of Cardiology, Heart Center Leipzig at Leipzig University, Leipzig, Germany; Department of Cardiology, University Medical Center of the Johannes Gutenberg University Mainz, Langenbeckstr. 1, 55131 Mainz, German Center for Cardiovascular Research - Partner Site Rhine-Main; Department of Cardiology, Heart Center Leipzig at Leipzig University, Leipzig, Germany; Department of Cardiology and Angiology, Medical Center - University of Freiburg, Faculty of Medicine, University of Freiburg, Freiburg, Germany; Department of Cardiology, University Medical Center of the Johannes Gutenberg University Mainz, Langenbeckstr. 1, 55131 Mainz, German Center for Cardiovascular Research - Partner Site Rhine-Main; Department of Cardiology, Heart Center Leipzig at Leipzig University, Leipzig, Germany; Department of Cardiology, Heart Center Leipzig at Leipzig University, Leipzig, Germany

**Keywords:** right ventricle, HFpEF, pressure-volume analysis

## Abstract

Heart failure with preserved ejection fraction (HFpEF) is a heterogeneous syndrome defined by diastolic dysfunction and limited therapeutic options, with increasing recognition of right ventricular (RV) involvement. Using invasive pressure-volume loop analysis, we assessed biventricular hemodynamics in lean and obese ZSF1 rats, a well-established rodent model of HFpEF. Obese rats exhibited significantly increased RV and left ventricular (LV) chamber stiffness, with a positive correlation between RV and LV stiffness constants, indicating biventricular diastolic dysfunction. RV end-systolic elastance was preserved, whereas LV contractility was increased. Despite elevated RV stiffness, myocardial fibrosis was unchanged, while RV and septal cardiomyocyte hypertrophy was significantly increased. These findings demonstrate that RV diastolic dysfunction in this HFpEF model is driven primarily by myocytic stiffening rather than fibrotic remodeling. Our data provide invasive haemodynamic evidence of RV involvement in HFpEF and further support the translational relevance of the ZSF1 rat model for studying biventricular HFpEF pathophysiology.

Heart failure with preserved ejection fraction (HFpEF) remains a significant clinical and scientific challenge, with limited effective therapeutic options. Its prevalence continues to rise, partly attributable to an aging population and common comorbidities such as hypertension, obesity, and metabolic disease. Advancing mechanistic insight and developing targeted therapies remain critical unmet needs. Preclinical research in HFpEF relies heavily on the assessment of *in vivo* physiological conditions, as the syndrome is primarily defined by functional haemodynamic alterations, namely changes in diastolic function, rather than distinct structural cardiac abnormalities. Subtle impairments in ventricular relaxation, compliance, and filling pressures are highly dynamic and influenced by loading conditions, making rigorous *in vivo* phenotyping essential for accurately modelling the disease and evaluating therapeutic efficacy. To date, only a limited number of *in vivo* models for HFpEF were developed, and each presents specific limitations in their ability to fully replicate the complex haemodynamic profile characteristic of the human condition.^1,2^ The current understanding of HFpEF pathophysiology recognizes it as a heterogenous and systemic disorder, involving not only the left ventricle (LV) and atrium but also the right ventricle (RV). Several mechanisms have been proposed to explain RV involvement, which may exacerbate the clinical manifestations and progression of HFpEF.

Invasive pressure-volume loop (PVL) analysis remains the reference standard for detailed haemodynamic assessment of cardiac function.^3^ However, to date, RV physiology has not been systematically evaluated using PVL analysis in rodent models of HFpEF, representing a gap in the preclinical characterization of this syndrome. In human HFpEF, major haemodynamic determinants of RV function include elevated end-diastolic RV pressures and increased chamber stiffness.^4^

Few preclinical models that recapitulate the hemodynamically defined HFpEF pathophysiology are available.^2^ The Zucker fatty and spontaneously hypertensive heart failure rate (ZSF1) serves as a widely used preclinical model for HFpEF and recapitulates major phenotypic traits of HFpEF, including concentric LV hypertrophy, preserved systolic function, diastolic dysfunction and exercise intolerance, as well as increased arterial stiffness, and insulin resistance.^2,5^

In this rodent model of HFpEF, lean (control) and obese ZSF1 rats (ZSF1-Lepr^fa^Lepr^cp^/Crl, Charles River, Kingston, USA; animal test reference number TVV 30/18) were studied at 20 weeks of age. Lean ZSF1 rats, that carry at least one functional leptin receptor allele exhibit normal metabolic and cardiac function and served as controls,^6^ whereas obese ZSF1 rats, homozygous for a leptin receptor mutation, develop obesity and HFpEF-like diastolic dysfunction. Animals were maintained on standard rat chow (RM1, SDS, Essex, UK) for 12 weeks starting at 8 weeks of age. For PVL analysis, a 1.49F pressure-volume catheter (Transonic Scisense Inc.) was introduced into the right ventricle via the right jugular vein and into the left ventricle via the carotid artery. In total, 12 lean and 12 obese ZSF1 rats were included in the analysis. PVL tracings were only included in the final analysis, if deemed suitable by two independent observers.

Body weights, heart weights and heart weight to tibia length ratios were significantly higher in obese rats [body weight: lean: 232 g (227 g; 254 g); obese: 467 g (450 g; 486 g); *P* < 0.01; heart weight: lean: 942 mg (896 mg; 963 mg); obese: 1355 mg (1323 mg; 1420 mg); *P* < 0.01; heart weight to tibia length ratio: lean: 25.7 (24.33; 26.10); obese: 36.70 (36.15; 38.50); *P* < 0.01].

Obese ZSF1 rats exhibited significantly increased RV stiffness compared with lean control rats. Specifically, the RV stiffness constant *β* was markedly elevated in obese rats [*Figure 1* panel A: lean ZSF1, *β* = 0.0016 (0.0013; 0.0026); obese, *β* = 0.0048 (0.0039; 0.0076); *P* < 0.01]. Likewise, the LV stiffness constant *β* was significantly greater in obese ZSF1 rats than in their lean counterparts [*Figure 1* panel B: lean, *β* = 0.0039 (0.0027; 0.0049); obese, *β* = 0.0132 (0.0093; 0.0251); *P* < 0.01]. A correlation between RV and LV stiffness constants was observed (*Figure 1* panel C: *R*² = 0.38, *P* < 0.05).

**Figure 1:**
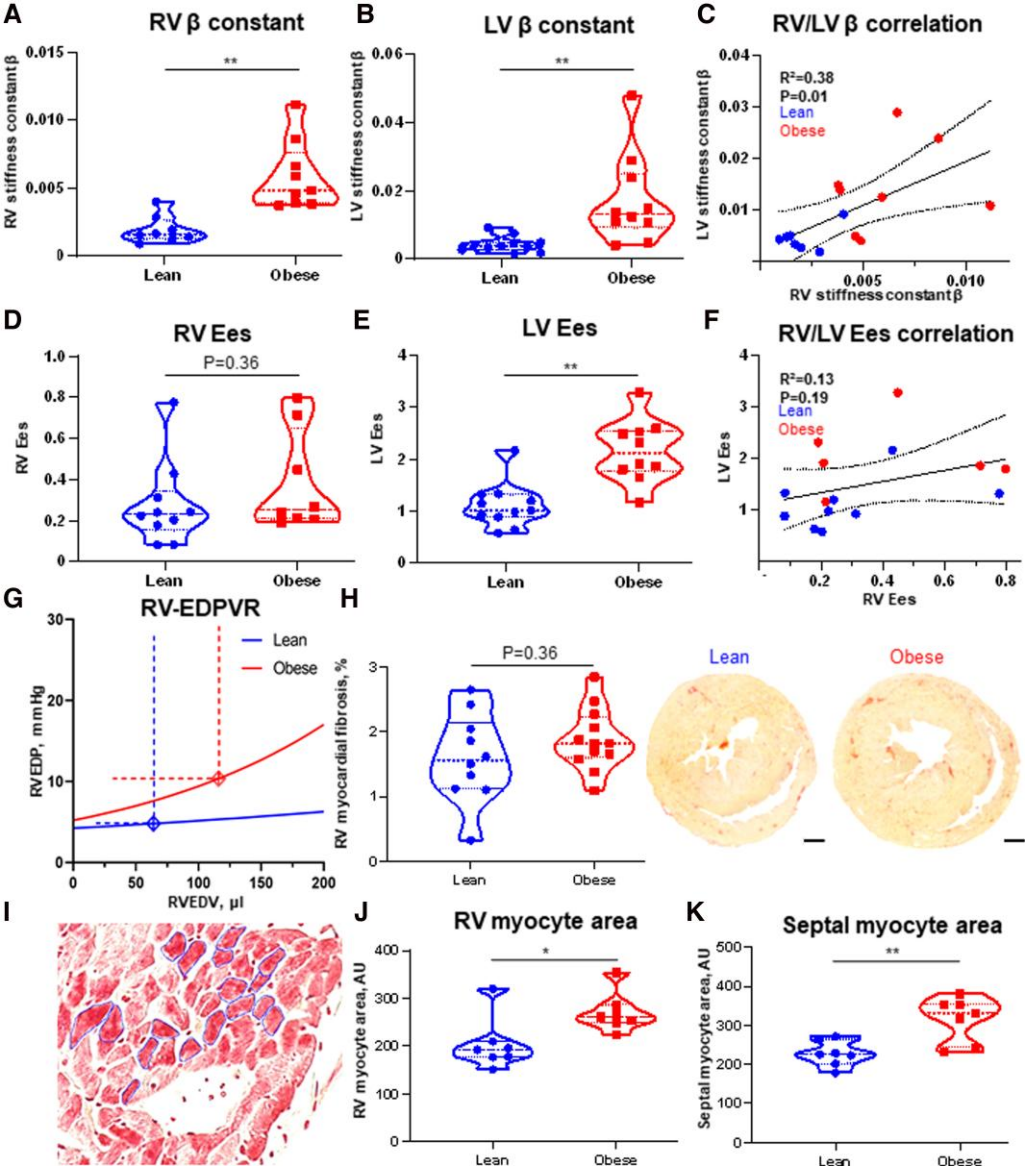
Right ventricular (RV) haemodynamic analysis in lean (*n* = 12) and obese (*n* = 12) diabetic Zucker fatty/spontaneously hypertensive heart failure F1 hybrid (ZSF1) rats: comparative analysis of the invasive PVL analysis derived beta constant, as a measure of ventricular stiffness, between lean and obese ZSF1-rats in the (*A*) RV, (*B*) left ventricle (LV) and (*C*) RV vs. LV. End-systolic elastance (Ees) in (*D*) the RV, (*E*) the LV and their correlation (*F*). (*G*) RV end-diastolic pressure-volume relationship (RV-EDPVR), (*H*) Amount of RV fibrosis determined by Picrosirius red staining with exemplary histological images, (*I*) Representative image of myocyte area measurement, (*J*) RV myocyte area, (*K*) septal, ventricular myocyte area; * < 0.05, ** < 0.01; bar in histological picture indicates 1 mm.

RV end-systolic elastance (Ees), defined as the slope of the end-systolic pressure-volume relationship (ESPVR), a load-independent measure of ventricular contractility that reflects the ventricle’s contractile adaptation to increased afterload, did not differ significantly between lean and obese ZSF1 rats [*Figure 1* panel D: lean, Ees = 0.23 (0.15; 0.34); obese, Ees = 0.25 (0.21; 0.65); *P* = 0.36]. In contrast, LV Ees was significantly increased in obese rats [*Figure 1* panel E: lean, Ees = 1.01 (0.09; 2.17); obese, Ees = 2.12 (1.76; 2.54); *P* < 0.01]. No significant correlation was found between RV and LV Ees values (*Figure 1* panel F: *R*² = 0.13, *P* = 0.19).

The RV end-diastolic pressure-volume relationship (EDPVR) in obese rats was steeper and left-shifted (*Figure 1* panel G), indicating increased RV chamber stiffness and impaired diastolic function.

Interestingly, RV myocardial fibrosis, quantified by picrosirius red staining, did not differ significantly between the groups [*Figure 1* panel H: lean, 1.6% picrosirius-positive area (1.1%; 2.1%); obese, 1.8% (1.6%; 2.2%); *P* = 0.31]. However, RV myocyte area, a marker of hypertrophy, was significantly larger in obese rats [*Figure 1* panel I, J: lean, 192 arbitrary units (AU) (177; 320); obese, 261 AU (249; 287), *P* < 0.05]. Similarly, septal myocyte area was greater in obese rats compared with lean controls [K: lean, 226 AU (202; 265); obese, 332 AU (244; 354), *P* < 0.01].

Despite technical challenges, comprehensive assessment of RV hemodynamics is essential for a full understanding of HFpEF, given the increasingly recognized role of biventricular involvement in disease progression. Here, we provide invasive haemodynamic evidence of RV diastolic dysfunction in a well-established *in vivo* HFpEF model. Obese ZSF1 rats exhibited markedly increased RV chamber stiffness and impaired diastolic properties, closely resembling key features observed in human HFpEF. The correlation between RV and LV stiffness supports the presence of shared pathophysiological mechanisms underlying biventricular diastolic dysfunction. Of note, increased RV stiffness occurred in the absence of enhanced myocardial fibrosis, indicating that extracellular matrix remodeling is unlikely to be the primary driver. Instead, significant RV myocyte hypertrophy points to myocytic stiffening as a major contributor.^7^ Our study corroborates prior human data, provides new mechanistic insight into RV involvement in HFpEF, and further validates the ZSF1 rat as a translationally relevant *in vivo* model that captures key biventricular features of HFpEF.

## Data Availability

The data underlying this article are available from the corresponding author upon reasonable request.

## Lead author biography

Florian Schlotter, MD, is a cardiologist and physician-scientist at the Centre for Cardiology, University Medical Centre Mainz, where he serves as Senior Consultant. He received his medical training at the University of Leipzig and completed research fellowships at Brigham and Women’s Hospital, Harvard Medical School. Dr. Schlotter is board-certified in internal medicine and cardiology, with additional qualifications in intensive care medicine and heart failure. His research focuses on the pathophysiology of heart failure and valvular heart disease, integrating invasive hemodynamics and translational models. He has published over 45 peer-reviewed articles and received multiple national and international research awards.
